# Use of the lung ultrasound score in monitoring COVID-19 patients: it’s time for a reappraisal

**DOI:** 10.1186/s13054-021-03483-y

**Published:** 2021-02-03

**Authors:** Luigi Vetrugno, Daniele Orso, Cristian Deana, Flavio Bassi, Gianmaria Cammarota, Tiziana Bove

**Affiliations:** 1 Department of Medicine, University of Udine, via Colugna n° 50, 33100 Udine, Italy; 2 Anesthesia and Intensive Care Medicine Department, ASUFC University-Hospital of Central Friuli, ASUFC, P.le S. Maria della Misericordia n° 15, 33100 Udine, Italy; 3 Department of Medicine and Surgery, University of Perugia, Perugia, Italy

To the Editor,

The lung ultrasound score (LUS)—as far as the literature reports—provides an overall rating of pulmonary aeration loss through the examination of 12 specified thoracic regions [1]. The level of aeration loss of each examined region is rated from 0 (absence of B lines) to 3 (lung consolidation), and the sum of these ratings constitutes the overall LUS, which can thus range from a minimum of zero to a maximum of 36 [1]. In non-COVID-19 patients with acute respiratory distress syndrome (ARDS), the LUS correlates with disease severity and mortality [1]. In COVID-19-related ARDS, a number of studies have assessed the role of the LUS in severity prediction and monitoring the response to treatment. Lung ultrasound is a quick- and easy-to-learn medical technique, rendering the LUS an easily accessible tool. The median time required for an expert operator to obtain a LUS is just 5 min. Ji and collaborators investigated the validity of using the LUS as a tool for monitoring the clinical progress of 280 COVID-19 patients [2]. The study confirmed their modified LUS (which generated an overall LUS scale of 0–60 by incorporating a score for pleural abnormalities [scale 0–2] for each of the 12 regions) to offer high prognostic accuracy (sensitivity and specificity both > 90%). Here, the authors proposed a cutoff value > 12 to predict an adverse outcome. Lichter et al., on the other hand, in their study of critically ill COVID-19 patients report an optimal cutoff value of 18 on the 0–36 scale for predicting adverse outcome, with a reported sensitivity of 62% and a specificity of 75% [3], whereas Zhu et al. report a sensitivity of 81% and a specificity of 96% with a cutoff value of 7 [4]. However, the study by Ji et al. [2] is difficult to compare with other studies in the literature for the following two reasons: first, they used a modified LUS scale (note a recent international expert consensus on the use of multi-organ point-of-care LU in COVID-19 adopts the scale range of 0–36 [5] and does not consider the pleural line artifact); second, the patients in the study by Ji et al. appear less critically ill than those in other studies, as evidenced by the fact that 88% of patients had an average value of PaO_2_/FiO_2_ greater than 300 mmHg.

## Data Availability

Not applicable.
